# Remote Delivery of the Cuidándome Telehealth Intervention for Self-Management of Depression and Anxiety Among Latina Immigrant Women: Randomized Controlled Trial

**DOI:** 10.2196/52969

**Published:** 2024-01-08

**Authors:** Carmen Alvarez, Subhash Aryal, Elizabeth Vrany, Maria Jose Sanchez R, Rosalphie Quiles, Lia Escobar-Acosta, Felicia Hill-Briggs

**Affiliations:** 1 School of Nursing University of Pennsylvania Philadelphia, PA United States; 2 Institute of Health System Science Feinstein Institutes for Medical Research Department of Medicine, Zucker School of Medicine New York, NY United States; 3 Department of Health Promotion College of Public Health University of Nebraska Medical Center Omaha, NE United States; 4 Division of Geriatric Medicine and Gerontology Johns Hopkins University School of Medicine Baltimore, MD United States; 5 Johns Hopkins School of Nursing Baltimore, MD United States

**Keywords:** Latina immigrant, mental health, depression, anxiety, problem-solving, intervention study, trauma-informed, depressive, Latinx, Latin, Latino, Latina, Hispanic, Spanish, immigrant, immigrants, survivor, child, children, childhood, trauma, traumatic, adverse, telehealth, telemedicine, eHealth, digital health, feasibility, acceptability, randomized, controlled trial, controlled trials, mobile phone

## Abstract

**Background:**

Growing evidence suggests that Latina immigrant survivors of adverse childhood experiences (ACEs) are at increased risk for developing and remaining with either depression or anxiety or both symptoms. This study examined the feasibility and acceptability of a telehealth intervention—Cuidándome (quee-DAN-doh-meh, “taking care of myself”). Cuidándome is a 10-week, patient-centered, trauma-informed intervention delivered by a trained facilitator that promotes self-management of depression and anxiety symptoms through improved problem-solving skills and strategies.

**Objective:**

The aim of this study was to examine the feasibility and acceptability of Cuidándome delivered remotely (via Zoom) with Latina immigrant ACE survivors with either depression or anxiety or both symptoms. We also estimated the effect sizes associated with the intervention on decreasing depression and anxiety symptoms and improving social problem–solving styles.

**Methods:**

We evaluated Cuidándome using a randomized controlled trial design. Latina immigrants (N=47) who had experienced at least 1 ACE and had at least mild depression or anxiety symptoms were randomized to Cuidándome or a comparison group delivered by trained facilitators. We assessed for changes in depression and anxiety symptoms as well as social problem–solving styles at baseline, post intervention, and 3- and 6-month follow-up.

**Results:**

Analyses indicated significant decreases over time within both Cuidándome and comparison groups for depression and anxiety symptoms and maladaptive problem-solving. The intervention effect was largest for anxiety; at 6-month follow-up, Cuidándome participants had significantly lower anxiety scores than the comparison group. In addition, we observed a greater average point reduction in depression symptoms at 6 months among Cuidándome participants (5.7 points) than in the comparison group (3.7 points).

**Conclusions:**

A mental health program delivered via Zoom by a trained facilitator was feasible and acceptable to Latina immigrant women and can be beneficial for reducing anxiety and depression symptoms. More research is needed to assess the effectiveness of Cuidándome among a powered sample size of Latina immigrants.

**Trial Registration:**

ISRCTN Registry ISRCTN16668518; https://www.isrctn.com/ISRCTN16668518

## Introduction

### Background

Latina immigrant survivors of adverse childhood experiences (ACEs) are at increased risk of poor long-term health outcomes, as mental health disorders often go untreated in this population [1]. ACEs are a spectrum of adversities that occur in 18 years and younger of age and include physical and sexual abuse, family dysfunction such as living with an adult with mental illness, experiencing or witnessing community violence (eg, stabbing and shooting), and experiencing or witnessing violence perpetrated by an organized group (eg, gang violence and police or military brutality) [2]. These types of experiences are established risk factors for anxiety and depressive disorders in adulthood [1,3]. The United States has seen historic levels of immigrants from Central America—many fleeing from different types of adversity, trauma (eg, natural disasters, pervasive community, and political violence), and limited opportunity for socioeconomic advancement. Growing evidence suggests that foreign-born Latinos, particularly those from countries with high risks of community and political violence, experience high rates of early childhood adversity that are associated with poorer mental health outcomes [3]. When compared to the general US population and Latino immigrant men, Latina immigrants report significantly higher rates of multiple types of ACEs [1].

Despite the high burden of adversity and depression and anxiety symptoms, multiple barriers impede Latina immigrants’ access to mental health services. System-level barriers such as lack of health insurance and lack of language-concordant services are common barriers to accessing mental health services [4]. Despite the growth of the Latino population in the United States, there has been a decline in mental health services offered in Spanish [5]. In addition, while evidence-based psychological treatments are the recommended first-line treatment for mild to moderate depression [6], they are becoming less available in primary care settings [7]. Implementation of evidence-based psychological treatment services in Latino-servicing health care settings also remains a challenge. These limitations make it difficult for Latino immigrants who prefer psychotherapy to pharmacotherapy to access the mental health care they need [8,9]. Studies targeting Latinos in primary care settings have often used licensed personnel as interventionists; however, the sustainability of providing such services in low-resource settings is questionable. To address these barriers, mental health experts recommend expanding access to behavioral health services by providing them outside of specialized settings, using telehealth services [10], and rigorously training and supervising unlicensed personnel (such as community health workers) to deliver high-quality services [11].

Teletherapy and the use of paraprofessionals both show promise in increasing acceptability and engagement in the treatment of Latino immigrants experiencing depression. Among Latinos, adherence to teletherapy sessions was higher (>80%) compared to in-person sessions (42%-80%) [12,13]. Despite the methodological limitations, paraprofessional-led interventions have demonstrated improvements in depression symptoms among Latinos [14,15]. Given the shortage of behavioral health professionals, delivering mental health care through paraprofessionals or community health workers is a promising strategy for increasing access for underserved populations [11,16].

Community health workers are also attuned to the needs of the communities they serve and have feasible solutions. Community health workers who serve Latino populations acknowledge (1) the need for more mental health services, (2) the training for community health workers to better meet this need, and (3) the use of teleservices to make care more accessible [17]. Community health workers have also proposed group support for addressing mental health needs. Indeed, the group format helps to reduce feelings of isolation and shame as participants hear from others who have similar life experiences with trauma and depression and anxiety symptoms [18]. Further, group support maximizes the community health workers’ reach as multiple individuals can be served and supported by each other.

Problem-solving is an established evidence-based approach for managing depression and anxiety symptoms. Social problem-solving refers to the cognitive behavioral process used to cope with life stressors [19]. According to problem-solving theory, coping with stressors involves two independent components: (1) problem orientation and (2) problem-solving style [20]. Problem orientation refers to one’s general cognition and attitudes when faced with a problem; this process is also framed by past experiences and self-appraisal about problem-solving ability. Problem-solving style refers to cognitive behavioral activities people use to cope with or manage stressful situations and include rational problem-solving (RPS; systematic and deliberate application of problem-solving skills), impulsive-careless style (ICS; impulsive approaches to problems), and avoidance style (AS; procrastination and avoiding addressing the problem) [21,22]. Effective social problem-solving involves identifying barriers to practicing recommended behaviors and brainstorming strategies to overcome barriers [23].

Among Latina immigrant women, ACE survivors had lower self-confidence in stress management compared to women who did not report ACEs [3]. In addition, experiencing more types of adversity was negatively associated with overall social problem–solving skills and positively associated with negative problem orientation (NPO) and AS [3]. Understanding and overcoming barriers through problem-solving underscore the importance of trauma-informed care, in which trauma survivors are supported in understanding how childhood adversities contribute to mental and physical health and reducing negative self-evaluations that impact problem-solving styles [24]. To date, the most widely used and evaluated psychological intervention among Latino immigrants is cognitive behavioral therapy, and the established benefit to Latina immigrants is based on 3 randomized controlled trials with limited generalizability [25]. Randomized controlled trials testing problem-solving therapy for decreasing depression symptoms among Latina immigrants showed clinically significant reductions in symptoms up to a year postintervention when compared to pharmacotherapy [26,27]. In summary, there remains a need to expand the portfolio of effective mental health interventions to maximize reach and enhance responsiveness to diverse needs among Latina immigrants.

### This Study

Given the lack of mental health services for Latina immigrants and the evidence for problem-solving and trauma-informed care, we developed Cuidándome (quee-DAN-doh-meh, “taking care of myself”). Cuidándome is a 10-week, culturally appropriate, trauma-informed, group-based intervention delivered once a week by a trained facilitator that promotes self-management of depression and anxiety symptoms through improved problem-solving skills and strategies. Multiple strategies were used to provide a trauma-informed intervention, including training the research team in therapeutic communication, screening and education about ACEs and their impact on health, and creating a safe and trusting environment for participants to work through their barriers for implementing useful strategies for depression and anxiety symptom management. Details of the adaptation and development process for Cuidándome are documented elsewhere [28]. The aims of this study were to (1) examine the feasibility and acceptability of Cuidándome delivered remotely (via Zoom; Zoom Video Communications) with Latina immigrant ACE survivors with either depression or anxiety or both symptoms and (2) estimate the effect sizes associated with the Cuidándome intervention on decreasing depression and anxiety symptoms and improving social problem–solving styles. We hypothesized that compared to the comparison group, the intervention group would report lower depression and anxiety symptoms, higher positive problem orientation (PPO) and RPS, and lower NPO, AS, and ICS at postintervention and at 3- and 6-month follow-up.

## Methods

### Recruitment

We recruited participants over 2 weeks in July 2021. Both active and passive strategies were used to recruit participants. Actively, we developed a database of Latina immigrants with a prior study [3] and contacted these women to assess for eligibility and participate in this study if interested. We also shared the study flyer with community health workers in the area, who distributed the flyer within their networks, including placing study flyers inside bags of food that were being donated during a food drive. Our passive strategies included posting flyers at laundromats and grocery stores in Latino-concentrated neighborhoods. Women who were interested in participating in the study texted or called the research phone. A bilingual research assistant obtained informed consent and established eligibility over the phone for all women verbalizing interest in participating in the study.

**Figure 1:**
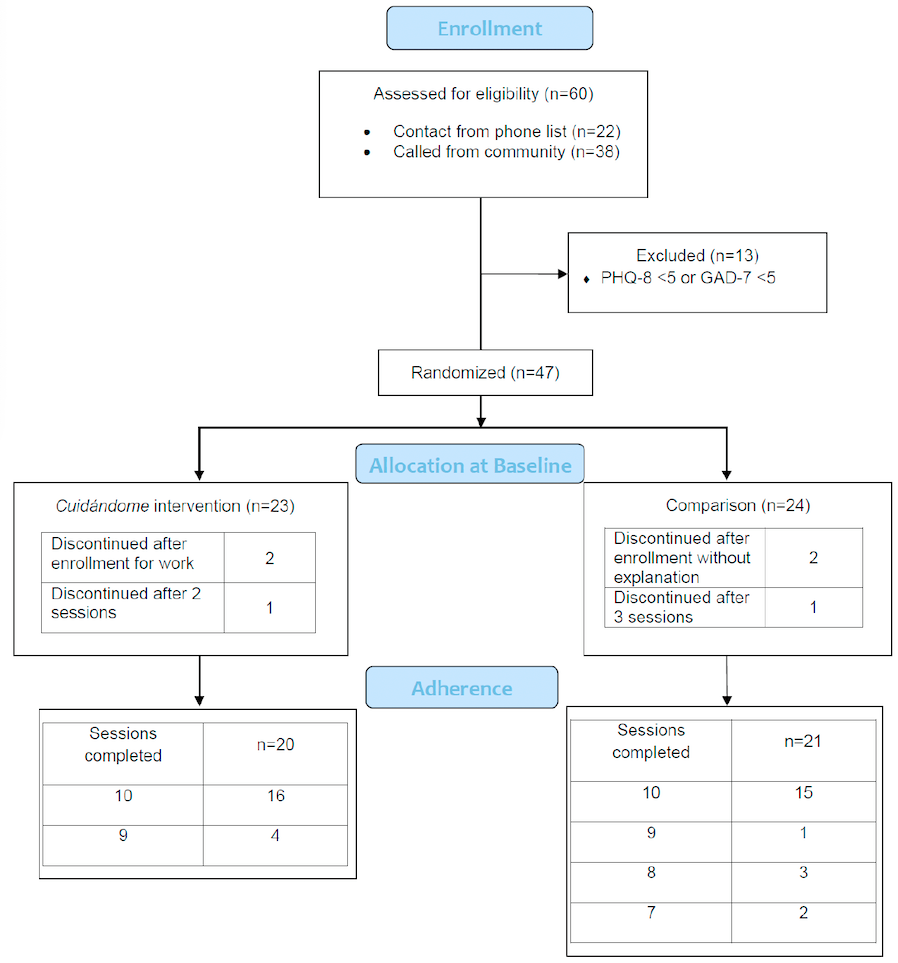
CONSORT flowchart of participant enrollment, allocation, and adherence. CONSORT: Consolidated Standards of Reporting Trials; GAD-7: Generalized Anxiety Disorder-7; PHQ-8: Patient Health Questionnaire-8.

Establishing eligibility included the completion of a baseline study questionnaire (including demographic information and assessments for depression and anxiety symptoms) to verify eligibility for the study. Eligibility criteria included (1) being ≥18 years, (2) foreign-born (or born on the island of Puerto Rico), (3) self-identify as a Latina, (4) self-report of ≥1 ACE, (5) ability to understand and speak Spanish, and (6) have a score of ≥5 on the Patient Health Questionnaire-8 (PHQ-8)—an assessment for depression symptoms [29] or ≥5 on the Generalized Anxiety Disorder-7 (GAD-7)—an assessment for anxiety symptoms [30]. We excluded women currently enrolled in another study about mental health (to limit potential confounding or carryover effects), and we excluded women who reported being pregnant (given that pregnancy can contribute to depression symptoms). Figure 1 displays the CONSORT (Consolidated Standards of Reporting Trials) diagram, participant enrollment, and retention (Multimedia Appendix 1).

### Ethical Considerations

All study procedures were approved by the Johns Hopkins University Ethics Review Board (IRB00287200). Oral consent was obtained from all participants in Spanish over the phone by the bilingual research assistants (native proficiency). To secure and protect all participant information, all data were collected and directly entered into REDCap (Research Electronic Data Capture) hosted at Johns Hopkins University. Only select research team members could access these data. All data were deidentified prior to export to SPSS (version 28; IBM Corp) for data analysis. Given the cost associated with data use, we compensated our participants up to US $190 for study participation (US $15 per session attended) and completion of all follow-up study questionnaires.

### Procedures

Women who provided consent were found eligible for the study (based on the baseline study questionnaire), and women who agreed to enroll in the study were randomized to receive either Cuidándome or educational content from a health promotion manual designed in Mexico [31]. Randomization was stratified based on ACE score so that one group would not have more people with higher average ACEs than the other. After completion of the baseline questionnaire and randomization, participants were told when their group sessions would begin, and participants were mailed the corresponding workbook for their group assignment. Based on input from community partners and the significant use of mobile phones and apps in the study population, we did not make computer or internet access a requirement for participation. For those who were not familiar with Zoom, a brief orientation was scheduled to explain how to use Zoom. All participants were encouraged to join the group sessions when they were scheduled; if a participant could not attend the group session, a make-up session would be scheduled with the participant where the facilitator would review content from the missed week prior to the next group session. This progression was particularly important because the sessions were designed to build on each other.

Study questionnaires were completed at baseline (T0) as part of the eligibility assessment and enrollment process, within 1 month post intervention (T1), and again at 3 months (T2) and 6 months (T3) post intervention. Trained bilingual research assistants who were not involved in the intervention delivery administered the study questionnaire (see “Study Questionnaire” section for descriptions of items and measures) to participants via phone and entered responses into a secure REDCap database. Based on our experience and prior evidence, many low-income Latino immigrants rely on their smartphones for internet access, particularly if they do not subscribe to broadband services [32]. Our retention efforts included 3 weekly reminders via SMS text message for joining the Zoom sessions, mailing participants a Cuidándome bookmark and a booklet of poems.

### Intervention

Table 1 provides the content overview for the intervention and comparison groups. To summarize, Cuidándome facilitates the learning and practice of systematic problem-solving through identification of the problem, generation of potential solutions, selection of the best solution, and implementation of the identified plan. Given participants’ history of trauma, the intervention sessions start with content about mental health and how ACEs, as well as other types of adversity, can contribute to mental health symptoms and conditions in adulthood. The remainder of the sessions guides participants through 5 evidence-based self-management strategies for managing depression and anxiety symptoms and identifying solutions for the barriers (life activities and stressors) that get in the way of practicing the recommended strategies. The weekly sessions lasted approximately 1 hour. In addition to the facilitator, all participants had the Cuidándome workbook that provided structured templates for guiding participants through the session activities. The first session included a discussion about ground rules, including the importance of confidentiality and not sharing comments made within the group with people outside of the group. During each session, the facilitator encouraged group discussion and shared reflections and strategies for overcoming challenges. The first 2 modules focus on psychoeducation and allow for discussion throughout. In the remaining modules, group learning through participant discussion is the priority; therefore, the facilitator presents the activity, guides participants through the exercises, and encourages discussion using vignettes and the workbook.

**Table 1:** Brief description of modules for Cuidándome and comparison program.

Cuidándome	Comparison (health promotion group)
**1A: ACEs^a^, depression, anxiety, and PTSD^b^** ACEs and their associations with health Signs and symptoms of depression, anxiety, and PTSD Mental health stigma	**1: Physical activity** What is physical activity? Benefits of physical activity Suggestions for remaining physically active
**1B: Mental health and self-management strategies** Signs and symptoms (continued) Review and discussion of personal profile Self-management strategies for depression and anxiety	**2A: Healthy eating** Review and discussion of food groups Discussion of portion sizes Recommendations for healthy eating
**2: Overview of problem-solving** Identify behaviors for self-management of mental health Identify barriers to self-management Understand the steps in problem-solving approach	**2B: Healthy eating** Recommendations for health eating My BMI
**3: Taking control of stress and emotions (problem orientation)** Understand negative versus positive problem orientation and its impact on problem-solving Understand the relationship between emotions and behavior	**3: Mental health** What is mental health? Why is it important? What can influence your mental health?
**4: What makes a problem a problem? (problem identification)** Identify external and individual barriers to self-management Demonstrate knowledge of the problem-solving process	**4: Substance misuse** Addiction prevention Assessing alcohol consumption Smoking cessation
**5: Know thyself: set goals that fit your life (generating alternative solutions)** Understand the importance of identifying problems for appropriate goal setting Demonstrate an understanding of effective goal setting	**5: Chronic diseases** Prediabetes and diabetes Hypertension Hyperlipidemia
**6: Different ways to reach health goals: knowing yourself** Understand the importance of exploring multiple options for problem-solving	**6: Cancer screenings** Breast cancer risk factors and screening Cervical cancer screening
**7: That sounds good but does it work for me?** Understand one’s own values and priorities in decision-making and problem-solving Demonstrate understanding of the 4 problem-solving styles and the impact on problem-solving Identify rational problem-solving as the effective approach for solving problems	**7: Osteoporosis** What is osteoporosis? Prevention of osteoporosis
**8: Take action and know the signs** Acquire skills for attempting alternative solutions for solving problems Demonstrate awareness of signs that a solution is not working	**8: Respiratory illnesses** Prevention and control of communicable respiratory infections Prevention and control of noncommunicable respiratory illnesses
**9: Putting it all together** Demonstrate mastery of the rational problem-solving approach Articulate the problem-solving approach for the management of mood	**9: Review** What did you learn? What has helped you? What will you continue to do for your well-being?

^a^ACE: adverse childhood experience.

^b^PTSD: posttraumatic stress disorder.

### Comparison

Given our focus on Latina immigrant women with either depressive or anxiety or both symptoms, we opted to offer the comparison group some generic health education content rather than be waitlisted. The content for the comparison group came from a family health promotion manual from Instituto Mexicano del Seguro Social—the Mexican Institute for Social Security [31]. We selected this manual given our focus on a Spanish-speaking, immigrant Latina population. The content from Instituto Mexicano del Seguro Social was already in Spanish and culturally appropriate—particularly the nutrition content that referenced traditional foods and diets common to our participants. The comparison content included 1 session about mental health conditions in general, which did not overlap with the more detailed Cuidándome training*.* Delivery of the comparison content mirrored the format of the intervention group: 1-hour weekly sessions delivered via Zoom.

### Group Facilitators and Intervention Fidelity

The intervention facilitator is a bilingual, Latina immigrant with a bachelor degree, who was trained in problem-solving therapy by our expert clinical psychologist and who received ongoing support and guidance. The facilitator of the comparison group was an experienced registered nurse with a master in health education and expertise (over 15 years of experience) in facilitating health promotion groups for Latina immigrant women. Aside from training on human participants, the nurse for the comparison group did not receive any specific training but was oriented to the purpose of the comparison group and provided with the corresponding workbook content. All Zoom sessions were audio recorded and reviewed after the sessions by the principal investigator to assess for client-centeredness (eg, showing empathy and encouraging autonomy) in both groups and to determine if the Cuidándome facilitator followed the facilitator script and demonstrated a problem-solving approach (eg, guiding participants to targets for change and focusing on positive action).

### Study Questionnaire

#### Demographic Characteristics

The study questionnaire included questions about demographic characteristics: age, relationship status, children, nativity, length of time in the United States, educational attainment, and employment status. Items about demographic characteristics were only administered at baseline.

#### Adverse Childhood Experiences

The Adverse Childhood Experiences-International Questionnaire was used, at baseline only, to assess for occurrence (eg, “Did you live with a household member who was a problem drinker or alcoholic, or misused street or prescription drugs?” “Yes” or “No”) and frequency (eg, “Did a parent, guardian or other household member hit or cut you with an object, such as a stick (or cane), bottle, club, knife, whip etc.” “Many times,” “A few times,” “Once,” or “Never”) of different types of adversities that occurred in the age of 18 years and younger [33]. In addition to items that inquired about the traditional ACEs (eg, physical and emotional neglect), the Adverse Childhood Experiences-International Questionnaire also assesses for the types of adversity such as bullying and experiencing or witnessing community violence. Items about child marriage were not included in our assessment because we have not identified this experience as a significant part of our population’s history. We dichotomized item responses based on the presence or nonzero frequency of an experience (yes=1 and no=0) and summed all dichotomized item responses for a total score; higher scores indicated experiencing more types of adversity. This tool has been validated with Latina immigrants [3,34].

#### Primary Outcome Variables

##### Depression

We used the PHQ-8 to assess the frequency (0=not at all to 3=nearly every day) of depression symptoms during the last 2 weeks [29]. Item responses are summed for a total score (range 0-24), with higher scores indicating greater severity of symptoms. The PHQ-8 has been validated among Latina immigrants and demonstrated good reliability with our sample (α=.83).

##### Anxiety

We used the GAD-7 to assess the frequency (0=not at all to 3=nearly every day) of anxiety symptoms during the last 2 weeks [30]. Item responses are summed for a total score (range 0-21), with higher scores indicating greater severity of symptoms. The GAD-7 has also been validated among Latina immigrants and demonstrated good reliability with our sample (α=.76).

##### Social Problem-Solving

We assessed social problem–solving styles using the Social Problem-Solving Inventory-Revised (SPSI-R) [20]. The items assess attitude toward challenges as well as one’s tendencies and approach for managing stressors in everyday life. Items present different styles of thinking and reactions to scenarios to which participants report how accurately the statement reflects their attitudes or behaviors to challenges (0=not at all true of me to 4=extremely true of me). The SPSI-R assesses for (1) problem orientation and (2) problem-solving style. Problem orientation refers to one’s disposition and attitude toward a problem. People with a PPO perceive problems as solvable challenges and are optimistic and confident in their ability to manage the problem; higher scores on the PPO subscale indicate greater confidence and optimism for solving problems. People with an NPO tend to perceive problems as a threat and are less confident in their ability to address the problem; higher scores on the NPO indicate less confidence in their ability to address problems.

Three problem-solving styles are assessed with the SPSI-R: RPS, ICS, and AS. When faced with challenges, people who practice RPS tend to think through multiple solutions and intentionally implement the optimal approach; higher scores on the RPS subscale indicate higher levels of RPS. The ICS is used to describe the tendency to act on the first option that comes to mind rather than consider multiple solutions; higher scores on the ICS subscale indicate greater impulsivity when addressing problems. The AS describes the practice of procrastination or avoiding addressing a problem; higher scores on the AS subscale indicate greater tendency for practicing avoidance for addressing problems. Each subscale was added for a sum score. To obtain a total social problem–solving score, the subscales are calculated (maladaptive styles negatively impact the total score social problem–solving score) using the prescribed formula [20]. These assessments have been used among Latina immigrants and demonstrated good reliability (α=.74).

### Statistical Analysis

Descriptive statistics (frequencies, means, and SDs) for all participant demographics and outcome variables were calculated. We tracked the number of sessions completed for each participant as an indicator of acceptability and asked participants if and how the intervention helped them at the end of the 10 weeks. The proportion of interested participants who consented and were screened as eligible to be in the study was also computed to help inform feasibility. We conducted independent *t* tests to assess differences in outcome variables (depression, anxiety, and social problem–solving styles) between the intervention and comparison groups at baseline and the follow-up time points (data not shown). In addition, paired *t* tests were used to compare the differences in means for the outcome variables from postintervention to 3- and 6-month follow-up time points (data not shown). For our primary analyses, mixed between-within participants’ ANOVA was used to examine differences in outcome variables between the intervention and comparison group, over time, from baseline to 6-month postintervention. We calculated effect sizes (Cohen *d*: small <0.50, medium ≥0.50 to <.80, and large ≥0.80) using the difference in outcome means for the different groups divided by the pooled SDs.

## Results

### Participant Demographics, ACEs, and Retention

Our sample included 47 Latina immigrants at baseline (Table 2) and 41 participants at all follow-up assessments. There were no significant differences between the intervention and comparison group at baseline.

**Table 2:** Participant characteristics^a^.

		Total sample^a^ (N=47)	Intervention group (n=23)	Comparison group (n=24)
Age (years), mean (SD)		35.72 (8.4)	36.78 (9.2)	34.7 (7.6)
**Relationship status,** **n (%)**				
	Single	8 (17)	3 (6)	5 (11)
	Married	16 (34)	8 (17)	8 (17)
	Living together (not married)	20 (43)	10 (21)	10 (21)
	Living apart (not married)	2 (4)	1 (2.1)	1 (2)
	Divorced	1 (2)	1 (2)	N/A^b^
**Children,** **n (%)**				
	None	4 (8)	3 (6)	1 (2)
	1-3	35 (75)	19 (41)	16 (34)
	4 or more	8 (17)	1 (2)	7 (15)
**Nativity,** **n (%)**				
	Mexico	11 (23)	5 (10)	6 (13)
	El Salvador	9 (19)	5 (11)	4 (8)
	Guatemala	5 (10)	2 (4)	3 (6)
	Honduras	12 (26)	4 (9)	8 (17)
	Other (Caribbean and South America)	10 (21)	7 (15)	3 (6)
Length of time in the United States, mean (SD)		10.48 (6.4)	11.3 (6.2)	9.7 (6.6)
**Education,** **n (%)**				
	Elementary school or less	9 (19)	3 (6)	6 (13)
	Some high school education	9 (19)	7 (15)	2 (4)
	High school graduate or more	29 (62)	13 (28)	16 (34)
**Employment status,** **n (%)**				
	Not employed	26 (55)	16 (34)	10 (21)
	Employed full-time	12 (26)	3 (6)	9 (19)
	Employed part-time	9 (19)	4 (9)	5 (11)
Adverse childhood experiences, mean (SD)		11.26 (4.8)	11.1 (5)	11.4 (4.6)
**Depression symptoms, baseline,** **n (%)**				
	None (<5)	6 (13)	3 (6)	3 (6)
	Mild (5-9)	18 (38)	8 (17)	10 (21)
	Moderate depression (10-14)	12 (26)	6 (13)	6 (13)
	Major depression, moderately severe (15-19)	6 (13)	4 (9)	2 (4)
	Major depression, severe (20-24)	5 (11)	2 (4)	3 (6)
**Anxiety symptoms, baseline,** **n (%)**				
	None (<5)	2 (4)	N/A	2 (4)
	Mild (5-10)	23 (49)	12 (26)	11 (23)
	Moderate (10-14)	14 (30)	9 (19)	5 (11)
	Severe (15-21)	8 (17)	2 (4)	6 (13)

^a^Baseline sample.

^b^N/A: not applicable.

The most common ACEs included community violence (n=39, 83%), witnessing violence in the home (n=39, 83%), emotional abuse (n=36, 77%), physical abuse (n=36, 77%), and being bullied (n=35, 75%). In addition, 55% (n=26) of the sample reported some form of sexual abuse (unwanted sex, fondling, and attempted sex).

Figure 1 displays participant enrollment, retention, and adherence to the group sessions. Of the participants we assessed for eligibility, most (n=38, 63%) were women who contacted the research team indicating their interest to participate. The other participants were selected from the database for a previous study. Attrition was low, with 6 women discontinuing participation primarily due to work schedules. All Cuidándome participants (n=20, 100%) completed at least 9 of the 10 total sessions, and 76% (n=16) of the comparison group completed 9 of the total 10 sessions.

### Depression and Anxiety and Social Problem-Solving

#### Overview

In Tables 3 and 4, we present the mean scores for depression, anxiety, and social problem–solving styles by study group (intervention and comparison) and time (baseline, postintervention, and 3- and 6-month follow-up). In Table 5, we compared for differences of change in scores between the intervention and comparison groups for depression, anxiety, and social problem–solving styles (interaction effect); compared the change in depression, anxiety, and social problem–solving styles over time within the groups (time main effect); and compared the 2 programs for changing depression, anxiety, and social problem–solving styles (intervention main effect).

**Table 3:** Group mean scores for depression, anxiety, and social problem–solving styles at baseline, postintervention, and 3- and 6-month follow-up.

Time point	Depression^a^				Anxiety^b^				Social problem–solving styles^c^		
	Intervention, mean (SD)	Comparison, mean (SD)	Effect size^d^	Intervention, mean (SD)		Comparison, mean (SD)	Effect size^d^	Intervention, mean (SD)		Comparison, mean (SD)	Effect size^d^
Baseline	10.75 (5.19)	10.19 (6.12)	N/A^e^	8.60 (3.36)		10.23 (5.36)	N/A	12.43 (2.94)		11.64 (2.60)	N/A
Postintervention	4.15 (3.04)	5.95 (4.26)	0.48	4.0 (2.99)		4.95 (3.29)	0.30	14.1 (1.41)		13.5 (2.67)	0.28
3-month follow-up	6.10 (4.66)	7.80 (6.16)	0.31	4.95 (2.62)		6.52 (5.56)	0.36	14.6 (2.35)		14.01 (3.31)	0.21
6-month follow-up	5.05 (2.95)	6.47 (4.58)	0.37	4.20 (1.93)		6.71 (5.01)	0.65	14.47 (1.91)		13.88 (3.13)	0.22

^a^Patient Health Questionnaire-8 [29].

^b^Generalized Anxiety Disorder-7 [30].

^c^Social Problem-Solving Inventory-Revised [20].

^d^Cohen *d:* difference in outcome means for the different groups divided by the pooled SDs.

^e^N/A: not applicable.

**Table 4:** Group mean scores for social problem–solving styles at baseline, postintervention, and 3- and 6-month follow-up.

	Negative problem orientation^a^				Avoidance style^a^				Impulsive-careless style^a^		
	Intervention, mean (SD)	Comparison, mean (SD)	Effect size^b^	Intervention, mean (SD)		Comparison, mean (SD)	Effect size^b^	Intervention, mean (SD)		Comparison, mean (SD)	Effect size^b^
Baseline	9.3 (4.29)	10.62 (4.90)	N/A^c^	4.8 (3.77)		5.57 (4.39)	N/A	5.95 (4.26)		7.66 (4.12)	N/A
Postintervention	6.1 (2.4)	6.42 (4.13)	0.10	2.1 (2.17)		4.42 (4.81)	0.62	4.65 (3.54)		5.19 (4.11)	0.14
3-month follow-up	5.45 (3.15)	7.66 (4.82)	0.54	2.40 (2.90)		3.67 (3.92)	0.36	4.55 (4.08)		7.1 (4.54)	0.59
6-month follow-up	4.85 (3.20)	6.42 (4.72)	0.38	1.65 (2.5)		4.0 (4.80)	0.61	4.45 (3.60)		6.10 (4.42)	0.40

^a^Social Problem-Solving Inventory-Revised [20].

^b^Cohen *d*: Difference in outcome means for the different groups divided by the pooled SDs.

^c^N/A: not applicable.

**Table 5:** Intervention and time effects on depression, anxiety, and social problem–solving styles.

	Depression		Anxiety		Social problem-solving		Negative problem orientation		Avoidance style		Impulsive-careless style	
	Wilks Λ	*F* test (*df*)	Wilks Λ	*F* test (*df*)	Wilks Λ	*F* test (*df*)	Wilks Λ	*F* test (*df*)	Wilks Λ	*F* test (*df*)	Wilks Λ	*F* test (*df*)
Intervention by time interaction	0.96	0.57 (3, 37)	0.96	0.48 (3, 37)	0.99	0.02 (3, 37)	0.92	1.1 (3, 37)	0.92	1 (3, 37)	0.89	1.5 (3, 37)
Time main effect	0.53	11.1 (3, 37)^a^	0.51	11.9 (3, 37)^a^	0.51	11.9 (3, 37)^a^	0.44	16.0 (3, 37)^a^	0.68	5.8 (3, 37)^b^	0.75	4.2 (3, 37)^c^
Intervention main effect	N/A^d^	0.91 (1, 39)	N/A	4.3 (1, 39)^a^	N/A	0.84 (1, 39)	N/A	1.6 (1, 39)	N/A	2.8 (1, 39)	N/A	2.2 (1, 39)

^a^*P*<.001.

^b^*P*<.005.

^c^*P*=.02.

^d^N/A: not applicable.

#### Depression

Based on the PHQ-8, depression levels decreased from baseline to postintervention for both groups and remained below baseline at 3 and 6 months (Table 3). There were small effect sizes (Cohen *d*) at each time point (postintervention (T1): *d*=0.48; 3-month follow-up (T2): *d*=0.31; and 6-month follow-up (T3): *d*=0.37; Table 3). Depression symptoms significantly decreased over time (time main effect) for both intervention and comparison groups (Wilks Λ=0.53; *F*_3,37_=11.1; *P*<.001; Table 5). However, when comparing the 2 groups, the change in depression symptoms over time was not significant (intervention by time interaction in Table 5). There was also no significant difference between the 2 programs in reducing depression symptoms (intervention main effect in Table 5); specifically, at each time point, there was no significant difference in depression symptoms between the groups.

#### Anxiety

Based on the GAD-7, anxiety levels also decreased from baseline to post intervention and remained below baseline through 6 months for both groups (Table 3). We estimated small (T1: *d*=0.30 and T2: *d*=0.36) and medium (T3: *d*=0.65) effect sizes for reduced anxiety symptoms (Table 3). The reduction in anxiety symptoms over time was significant, with both groups showing a reduction in symptoms across the follow-up time points (Wilks Λ=0.51; *F*_3,37_=11.9; *P*<.001; Table 5). There was also a significant difference in the reduction of symptoms between the 2 groups, where Cuidándome was demonstrated to be more effective than the comparison program for reducing anxiety symptoms (*F*_1,39_=4.3; *P*<.001).

#### Social Problem–Solving Styles

Overall social problem-solving increased from baseline to all 3 time points. Similar to depression and anxiety symptoms, the increase in social problem-solving over time was significant with both groups showing improvement (Wilks Λ=0.51; *F*_3,37_=11.9; *P*<.001). When comparing the 2 groups, the increase in social problem–solving scores over time was not significant (intervention by time interaction in Table 5). There was also no significant difference between the 2 programs for increasing social problem–solving scores (intervention main effect in Table 5). The ANOVA analyses indicated that neither program had a significant effect on PPO or RPS (data not shown).

### Negative Problem Orientation

NPO decreased from baseline to all 3 time points (Table 4). The reduction in NPO over time was significant for both groups (Wilks Λ=0.44; *F*_3,37_=16; *P*<.001; Table 5). Although there was change over time for both groups, this change did not differ by group (intervention by time interaction), and there was no significant difference in the effect of the 2 programs for reducing NPO (intervention main effect). However, the difference in NPO at 3-month follow-up between the 2 groups almost reached significance (Cuidándome: mean 5.45, SD 3.15 vs comparison: mean 7.66, SD 4.82; *P*=.09), and we estimated small (T1: *d*=0.10) and medium (T2: *d*=0.54 and T3: *d*=0.38) effect sizes (Table 4).

### Avoidance Style

ASs decreased from baseline to all 3 time points (Table 4). Similar to NPO, the reduction in AS over time was significant for both groups (Wilks Λ=0.68; *F*_3,37_=5.8; *P*<.005; Table 5). There was no difference in change over time between the 2 groups, and there was no significant difference in the effect of the 2 programs for reducing ASs; however, the difference in mean scores postintervention (Cuidándome: mean 2.1, SD 2.17 vs comparison: mean 4.42, SD 4.81; *P*=.06) and at 6-month follow-up (Cuidándome: mean 1.65, SD 2.5 vs comparison: mean 4.0, SD 4.80; *P*=.06) approached significance, where Cuidándome participants reported lower AS at these time points (Table 4). We estimated medium effect sizes (T1: *d*=0.62, T3: *d*=0.61) for the intervention.

### Impulsive-Careless Style

ICS decreased from baseline to all follow-up time points (Table 4). ICS decreased over time for both groups (Wilks Λ=0.75; *F*_3,37_=4.2; *P*=.02); however, the change in scores did not differ significantly between the groups, and neither program was more effective at reducing ICS scores (Table 5). Nonetheless, we observed lower mean scores for Cuidándome participants compared to the comparison group that approached significance at 3-month follow-up (Cuidándome: mean 4.55, SD 4.08 vs comparison: mean 7.1, SD 4.54; *P*=.07; Table 4). We calculated small (T1: *d*=0.14) and medium (T2: *d*=0.59 and T3: *d*=0.40) effect sizes for the intervention.

## Discussion

### Principal Findings

This study is one of the first to assess the feasibility and acceptability of a trauma-informed, problem-solving–based, self-management program delivered remotely for Latina immigrant ACE survivors with at least mild depression and anxiety symptoms. Our rapid recruitment (completed in 2 weeks), high attendance, and retention indicated that participants desired the program and found it acceptable. Based on the PHQ-8 means, participants in both groups were experiencing moderate levels of depression symptoms at baseline. Depression symptoms significantly decreased for both groups, with no significance in change between the intervention and comparison group. However, we observed lower depression scores among Cuidándome participants compared to the comparison group suggesting Cuidándome participants experienced fewer days with depression symptoms. Although both groups experienced improvements, Cuidándome participants on average reported a greater reduction in depression symptoms (5.7 points) compared to the comparison group (3.7-point reduction). For anxiety symptoms, Cuidándome was significantly more effective at reducing anxiety symptoms. On average, Cuidándome participants reported minimal to no anxiety symptoms at all follow-up time points compared to comparison group participants who on average reported mild symptoms. The effect sizes for depression and anxiety were small to medium, further supporting the beneficial impact of Cuidándome for these symptoms. Further study with a powered sample is needed to rigorously test the effectiveness of Cuidándome in this Latina immigrant population.

Contrary to our hypothesis, we did not see improvements in PPO or RPS—the components of social problem–solving that we anticipated Cuidándome would increase. Instead, we found that among Cuidándome participants, we observed lower scores for NPO and maladaptive problem-solving styles (avoidance and impulsive-careless). The goal setting and learning the problem-solving steps may have helped Cuidándome participants feel more inspired and empowered to address daily life challenges in order to pursue their goals. When discussing the benefits of Cuidándome, our participants shared that Cuidándome provided them with the steps for “how” to achieve their goals; this may have helped women have a more positive outlook on addressing challenges [28]. Women also shared that they felt a greater sense of confidence managing daily challenges and thinking through options before reacting to a situation. Regarding the lack of findings with the positive subscales, although we did not include assessments of social desirability, social desirability may have influenced participant responses and minimized the scales’ sensitivity to change. Further, we were not powered to identify statistically significant changes with any of our outcomes.

Other studies that have examined the effectiveness of problem-solving therapy for depression among Latinos have also identified improvements in depression symptoms [27,35]. However, this study is the first to show promising findings on anxiety as well as social problem–solving styles, which are the potential mechanisms of action for improving mental health outcomes.

We unexpectedly observed significant reductions in depression and anxiety symptoms in both groups. On review of the session recordings, we learned that the nurse facilitator for this group used both goal-setting and problem-solving (particularly brainstorming solutions) strategies in her sessions—particularly for the nutrition and physical activity sessions. At the end of these sessions, participants were encouraged to set a goal based on the session topic, and they discussed strategies for achieving those goals. Participants also exchanged contact information with fellow participants in the chat feature of Zoom. We did not assess social support, but it is possible that through these sessions, participants were able to expand their social network and increase social support, which is associated with lower depression and anxiety symptoms [36]. In addition, given that there were 2 sessions about physical activity and nutrition, comparison group participants may have increased their physical activity; indeed, increased physical activity is associated with a reduction in depression symptoms among Latina women [37] and other populations [38]. Finally, for all participants, we made ourselves available to connect them with community resources. Participants often called for information about where they could find health care services, work, and food. In our future work, we will assess whether change in social needs is associated with improved mental health.

Based on prior work, we surmise that the trauma-informed content and care from our research team were important contributors to retention. During the development phase of Cuidándome, the review of ACEs and their association with mental health conditions were the most time-consuming sessions because of participant engagement. Similar to findings by Kaltman et al [39] who also examined the feasibility and acceptability of an in-person, trauma-informed intervention, Cuidándome participants had positive reviews about the discussions on trauma, and they found it validating to learn that their current depression and anxiety symptoms could be related to early life adversities. Participants who were mothers felt inspired to engage with their children in a more positive way to not perpetuate the cycle of ACEs. Qualitative analysis of participant discussions during these sessions may provide more insight into participant responses to the trauma-informed content.

The success of this feasibility study may also be attributed to the intervention being offered remotely. Multiple structural (eg, documentation status) [40] and system-level barriers (eg, accessibility, health insurance, and language concordant services) make mental health services and care inaccessible for Latina immigrant women [41]. Cuidándome eliminated several of these macrolevel barriers—there was no need for participants to present themselves in any establishment with government-issued identification in order to obtain services, participants did not have to travel to a physical location, health insurance was not required, and the program was offered in Spanish. Using telehealth and trained personnel eliminated barriers that prevent marginalized groups from accessing a program that may be beneficial for mental health. More research with stakeholders is needed to determine how programs such as Cuidándome can be made more accessible and sustainable in community-based settings.

### Limitations

We acknowledge several limitations with this study. First, we sought to establish acceptability and feasibility and did not calculate a sample size a priori. Our relatively small sample size may explain the few statistically significant findings between Cuidándome and the comparison program. In addition, our sample represented women primarily in urban and suburban settings with access to broadband services. A larger sample size that includes some geographic diversity may yield more generalizable findings.

Despite the limitations, this work contributes to the body of literature highlighting specific useful strategies (telehealth and nonlicensed personnel) that can be used to expand access to mental health services for populations socially at risk and underserved populations. Nonlicensed personnel such as community health workers have successfully delivered mental health services in low-resource settings [16]. This work aligns with other studies demonstrating the acceptability and effectiveness of training nonlicensed personnel to deliver mental health interventions [39,42] as well as the use of a web-based platform for administering these programs.

### Conclusions

Our findings indicate that the Cuidándome intervention can improve depression and anxiety symptoms among Latina immigrant ACE survivors. Further, Cuidándome may also be beneficial for decreasing maladaptive behaviors (avoidance and impulsivity) associated with depression and anxiety symptoms. As the Latina immigrant population continues to grow, so should community-based mental health resources. More methodologically rigorous study of Cuidándome is needed; however, this study shows the promise of an intervention that leverages nonlicensed personnel and uses a web-based platform to increase the availability of a beneficial mental health program.

## Appendix

Multimedia Appendix 1 CONSORT e-HEALTH (V.1.6.1) checklist.

## Abbreviations

ACE: adverse childhood experience
AS: avoidance style
CONSORT: Consolidated Standards of Reporting Trials
GAD-7: Generalized Anxiety Disorder-7
ICS: impulsive-careless style
NPO: negative problem orientation
PHQ-8: Patient Health Questionnaire-8
PPO: positive problem orientation
REDCap: Research Electronic Data Capture
RPS: rational problem-solving
SPSI-R: Social Problem-Solving Inventory-Revised

## Data Availability

The data sets generated and analyzed during this study are not publicly available due to ethical considerations but are available from the corresponding author on reasonable request.
